# Supplementary material for: iNOS Deletion in Alveolar Epithelium Cannot Reverse the Elastase-Induced Emphysema in Mice
Source: Cells. 2022 Dec 28;12(1):125. doi: 10.3390/cells12010125 (PMC9818765; doi:10.3390/cells12010125)
Supplement: Supplementary file 1 [file cells-12-00125-s001.zip › cells-1974762-supplementary.pdf]

iNOS deletion in alveolar epithelium cannot reverse the elastase-induced emphysema in mice

Marija Gredic, Vinita Sharma, Stefan Hadzic, Cheng-Yu Wu, Oleg Pak, Baktybek Kojonazarov, Julia Duerr, Marcus A. Mall, Andreas Guenther, Ralph T. Schermuly, Friedrich Grimminger, Werner Seeger, Simone Kraut, Natascha Sommer and Norbert Weissmann

ONLINE SUPPLEMENT

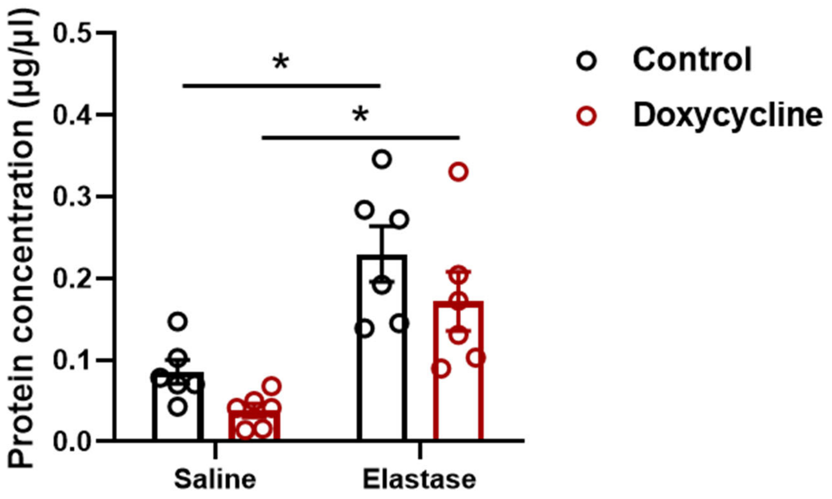

Supplemental Figure S1. Protein content in BALF of saline- or elastase-treated *iNos CCSP rtTA2s-M2 LC1* mice fed either with normal (Control) or doxycycline-containing (Doxycycline) chow, after the 12-week observation period, quantified using Bradford assay (n=6). Graph shows mean ± SEM. *$p < 0.05$. Two-way ANOVA (with Tukey´s multiple comparison post-hoc test) was used.
